# Host-antibody inductivity of virulent *Entamoeba histolytica* and non-virulent *Entamoeba moshkovskii* in a mouse model

**DOI:** 10.1186/s13071-019-3363-5

**Published:** 2019-03-13

**Authors:** Narumol Khomkhum, Somphob Leetachewa, Aulia Rahmi Pawestri, Saengduen Moonsom

**Affiliations:** 1 Department of Protozoology, Faculty of Tropical Medicine, Mahidol University, Ratchawithi, Bangkok, 10400 Thailand; 2 Center for Vaccine Development, Institute of Molecular Biosciences, Mahidol University, Nakhorn-Pathom, 73170 Thailand

**Keywords:** Amoebiasis, *Entamoeba histolytica*, *Entamoeba moshkovskii*, Pathogenicity, Antibody dependent enhancement

## Abstract

**Background:**

Despite similarities in morphology, gene and protein profiles, *Entamoeba histolytica* and *E. moshkovskii* show profound differences in pathogenicity. *Entamoeba histolytica* infection might result in amoebic dysentery and liver abscess, while *E. moshkovskii* causes only mild diarrhea. Extensive studies focus on roles of host immune responses to the pathogenic *E. histolytica*; however, evidence for *E. moshkovskii* remains scarce.

**Methods:**

To study differences in host-antibody response profiles between *E. histolytica* and *E. moshkovskii*, mice were immunized intraperitoneally with different sets of *Entamoeba* trophozoites as single species, mixed species and combinations.

**Results:**

Mice prime-immunized with *E. histolytica* and *E. moshkovskii* combination, followed by individual species, exhibited higher IgG level than the single species immunization. Mice immunized with *E. moshkovskii* induced significantly higher levels and long-lasting antibody responses than those challenged with *E. histolytica* alone. Interestingly, *E. histolytica*-specific anti-sera promoted the cytopathic ability of *E. histolytica* toward Chinese hamster ovarian (CHO) cells, but showed no effect on cell adhesion. There was no significant effect of immunized sera on cytopathic activity and adhesion of *E. moshkovskii* toward both CHO and human epithelial human colonic (Caco-2) cell lines. Monoclonal-antibody (mAb) characterization demonstrated that 89% of *E. histolytica*-specific mAbs produced from mice targeted cytoplasmic and cytoskeletal proteins, whereas 73% of *E. moshkovskii*-specific mAbs targeted plasma membrane proteins.

**Conclusions:**

The present findings suggest that infection with mixed *Entamoeba* species or *E. moshkovskii* effectively induces an antibody response in mice. It also sheds light on roles of host antibody response in the pathogenic difference of *E. histolytica* and *E. moshkovskii* trophozoites, and cell surface protein modifications of the amoebic parasites to escape from host immune system.

## Background

*Entamoeba histolytica* is an anaerobic pathogenic protozoan parasite that causes approximately 100,000 global deaths annually due to amoebiasis [1]. Disease symptoms range from mild diarrhea to severe bloody diarrhea with mucus as the parasite invades the intestinal epithelium [2]. After invading the intestinal *lamina propria*, this parasite enters the blood stream to reach other organs and results in abscesses, most commonly in the liver [3], and rarely in lungs [4] and brain [5]. The pathogenesis of *E. histolytica* starts with parasite adhesion at the large intestinal epithelium and secretion of cysteine proteases, leading to the degradation of host tissues. The secreted cysteine proteases play important roles in degrading gut mucosal IgA and circulating IgG, resulting in the ineffectiveness or failure of host immunity, thus inversely promote extra-intestinal infection of *E. histolytica* [6, 7]. In addition, the parasite-gut adhesion was shown to trigger host signal transductions through caspases 3-like cascade and caspases 8- and 9-independent manner [8]. These lead to apoptotic cell death, which were preferentially phagocytosed by the parasite. The interaction also stimulates production of pro-inflammatory cytokines, including interleukin (IL)-1, IL-6, IL-8, IFN-γ and tumor necrosis factor (TNF)-α, which consequentially promote tissue damages and severity of the disease [9, 10]. Inhibition of TNF-α has been proved to significantly reduce the inflammation and tissue destruction [11], while the absence of the anti-inflammatory cytokine IL-10 has been shown to result in increased severity of intestinal amoebiasis [12]. Thus, the manifestation of amoebiasis apparently happens through the parasite’s ability to activate cytokine-mediated cell deaths and manipulate the host immune system.

*Entamoeba moshkovskii* was previously considered as a non-pathogenic protozoan parasite, which was commonly found to co-occur in human stools collected from *E. histolytica* endemic areas, often leading to misdiagnosis of *E. histolytica* due to their mostly identical morphology [13, 14]. Despite being considered non-pathogenic, *E. moshkovskii* has been gradually reported as associated with diarrhea in humans and mice [15–17]. Recently, *E. moshkovskii* was reported to cause subcutaneous abscess in Indonesia [18]. Shimokawa et al. [16] showed that *E. moshkovskii* was able to cause symptoms, including weight loss, diarrhea and colitis in susceptible mice as is the case for *E. histolytica*. Furthermore, damages of the intestinal epithelium of *E. moshkovskii-*infected mice were observed due to host IFN-γ mediated cell apoptosis [17].

In the present study, we aimed to investigate the immunogenicity of *E. histolytica* and *E. moshkovskii* trophozoites through host-antibody response profiles as well as effect of the immunized sera on *Entamoeba* pathogenicity. We found that mouse immunization with mixed *Entamoeba* species was able to induce both specific IgA and IgG higher levels than single species. The effect of the immunized sera on cytopathic activity and host cell adhesion were investigated and the possible immune evasion and cell manipulating mechanisms by *E. histolytica* are discussed. Our findings may shed more light on *E. histolytica* pathogenicity, which can be of further benefit in the development of diagnosis modalities, treatment and vaccines for this parasite.

## Methods

### Mouse immunization with *Entamoeba* cells

Trophozoite cells of *E. histolytica* strain HM1: IMSS and *E. moshkovskii* strain Laredo, which were kindly provided by Professor Tomoyoshi Nozaki, Department of Biomedical Chemistry, Graduate School of Medicine, University of Tokyo, Japan, were axenically cultured in bis-iron serum (BIS) medium at 37 °C and 26.5 °C, respectively. Cells were harvested by placing culture tubes on ice for 10 min to detach the cells, followed by centrifugation at 200× *g* for 3 min at 4 °C with three washes using cold phosphate-buffered saline (PBS). Viable amoeba cells were counted using a hemocytometer by trypan blue exclusion (0.2% trypan blue). For studies of host-antibody response, BALB/c mice (3 mice/group; 12 mice in total) were immunized with 2 × 10^6^ cells of mixed species (1 × 10^6^ cells each of *E. histolytica* and *E. moshkovskii*) or 2 × 10^6^ cells of individual species (*E. histolytica* or *E. moshkovskii*) according to 4 immunization designs (group 1 mice received *E. histolytica* cells for 4 doses; group 2 mice received *E. moshkovskii* cells for 4 doses; group 3 mice received *E. histolytica* and *E. moshkovskii* cell mixture for 2 doses, followed by *E. histolytica* cells for 2 doses; group 4 mice received *E. histolytica* and *E. moshkovskii* cell mixture for 2 doses followed by *E. moshkovskii* cells for 2 doses). Immunization was performed intraperitoneally (IP) with two-week intervals. Whole blood was collected from the ventral tail vein before each immunization [19] and after the 4th boost for 2 weeks (B4: bleed 4) and 8 weeks (B5: bleed 5). Serum collected before the first immunization (pre-immunized serum) was used as a negative control for the baseline antibody level of each mouse.

### Monoclonal antibody (mAb) production

BALB/c mice (2 mice per set) were immunized with 2 × 10^6^ cells of *E. histolytica* and *E. moshkovskii* trophozoite for 3 doses followed by 2 doses of mixed *Entamoeba* cells (1 × 10^6^ cells each of *E. histolytica* and *E. moshkovskii*) in two-week intervals. Blood samples were collected from the tail vein before each immunization and tested for *Entamoeba*-specific antibodies. The mice were sacrificed and splenic B cells were fused with mouse myeloma cells using the standard hybridoma technique described by Moonsom et al. [19]. Hybridoma cells secreting *Entamoeba*-specific antibodies were screened by enzyme-linked immunosorbent assay (ELISA) with *Entamoeba* cell lysate proteins. Limiting dilutions were performed to obtain the mAb-producing cells.

### Preparation of cellular protein compartments

Axenically cultivated *E. histolytica* and *E. moshkovskii* trophozoite cells were harvested and washed with PBS, pH 7.4. Total cell lysate proteins were solubilized using mammalian protein extraction reagent (M-PER) (Thermo Fisher Scientific, Waltham, MA, USA). Cells were fractioned into cytoplasmic, membranous, nuclear and cytoskeletal portions using a Qproteome cell compartment kit (Qiagen, Hilden, Germany) following the manufacturer’s instructions. Protein profiles of cell fractions were analyzed on a 10%-gel using sodium dodecyl sulfate-polyacrylamide gel electrophoresis (SDS-PAGE) and protein concentration was determined using Bradford’s assay following the manufacturer’s instructions (Bio-Rad, Hercules, CA, USA).

### ELISA of anti-serum and mAbs with cellular fractions

Cellular protein fractions were immobilized onto the ELISA microplate for overnight at 4 °C. After blocking with 2% skim milk, culture supernatant or the immunized mouse serum containing primary antibodies; anti-*E. histolytica* (anti-Eh4), anti-*E. moshkovskii* (anti-Em6) and anti-pan-human *Entamoeba*; *E. histolytica*, *E. moshkovskii* and *E. dispar* (anti-Ehmd4) mAb or 1:5000 diluted pre-immunized serum (a control) were applied. The reactions were incubated for 1 h at room temperature. *Entamoeba* binding partner-mAb complexes were detected with 1:5000 diluted anti-mouse immunoglobulins (Thermo Fisher Scientific), anti-mouse IgG (Sigma-Aldrich, St. Louis, MO, USA) and anti-mouse IgA (Sigma-Aldrich) conjugated with horse radish peroxidase (HRP) enzyme (Thermo Fisher Scientific) for 1 h. The colorimetric signal was developed using 3,3′,5,5′-tetramethylbenzidine (TMB) substrate (Thermo Fisher Scientific) and quenched with 1N hydrochloric acid (HCl). The color intensity was recorded at a wavelength of 450 nm using a Sunrise microplate reader (Tecan, Männedorf, Switzerland). The measurement was performed in triplicate and presented as means of optical density (OD) ± standard error of the mean (SEM).

### Immunofluorescent assay of specific anti-sera with *Entamoeba* cells

Axenically cultivated trophozoites of *E. histolytica* and *E. moshkovskii* were harvested and attached onto cell imaging slides (Eppendorf, Hamburg, Germany). The immobilized cells were fixed with 3.7% paraformaldehyde at room temperature for 10 min and permeabilized with 0.2% Triton X-100 in 1% bovine serum albumin (BSA)-PBS for 10 min. Non-permeabilized (incubated in 1% BSA-PBS) and permeabilized cells were reacted with the immunized mouse serum (1:1000) or culture supernatant containing mAb (1 mg/ml mAb) for 1 h. Antibody positive cells were detected with anti-mouse immunoglobulin G conjugated with FITC (1:60) and viewed by confocal microscopy (LSM 700; Carl Zeiss, Jena, Germany). All immunological reactions were performed in 1% BSA-PBS.

### CHO cytopathic and cell-adhesion assays of *E. histolytica* and *E. moshkovskii*

Chinese hamster ovary (CHO) cell lines were cultured in Roswell Park Memorial Institute (RPMI) medium supplemented with 10% fetal bovine serum (FBS) at 37 °C in a CO_2_ incubator. Cells were harvested by 0.1 mM ethylene diamine tetraacetic acid (EDTA)-PBS, pH 7.4 and washed 3 times with cold PBS. Viable trophozoite cells of *E. histolytica* (1 × 10^5^ cells) and *E. moshkovskii* (2 × 10^5^ cells) were pre-incubated with pre-immunized or immunized serum (1:5000 dilution in 10% FBS-RPMI medium) at 4 °C for 30 min. The *Entamoeba* cell-serum mixtures were then incubated with 2 × 10^5^ of CHO-cell suspension at a ratio of 1:5 for *Entamoeba* and CHO cells at 4 °C for an additional 1 h. CHO cells incubated with *Entamoeba* cells in culture medium or serum alone were used as controls. CHO cell adhesion was defined as an *Entamoeba* cell formed with at least three CHO cells [20, 21]. CHO-*Entamoeba* cell mixtures were stained with 500 nM propidium iodine (PI). PI positive CHO cells were counted under fluorescence microscopy (Observer Z1; Carl Zeiss) to assess the killing ability of *Entamoeba* cells. The assay was performed in triplicate and presented as percent amoeba cell adhesion ± standard error of the mean (SEM) or percent cell death with 95% confidence interval (CI).

### *Entamoeba histolytica* and *E. moshkovskii* adhesion to human epithelial Caco-2 cell line

*Entamoeba histolytica* or *E. moshkovskii* cells (2 × 10^5^) were pre-mixed separately with a pre-immunized and dose-4 immunized serum at a final dilution of 1:5000 in 250 μl of 10% FCS-Iscove’s Modified Dulbecco’s Medium (IMDM). The *Entamoeba* cell-serum mixture was incubated with a monolayer of Caco-2 cells (provided by Professor Kris Chadee, Department of Microbiology, Immunology and Infectious Diseases, University of Calgary, Canada) in a 24-well plate at the ratio of 1:1 of *Entamoeba*:Caco-2 cells at 37 °C for 15 min. The culture supernatant was removed and then the cells were washed twice with PBS. The adhered *E. histolytica*/*E. moshkovskii* cells were counted from at least 5 fields per well with 20× magnification using inverted microscopy (Observer Z1; Carl Zeiss). The experiment was performed in triplicate and presented as percent cell adhesion ± SEM.

### Statistical analysis

Statistical analyses of cytopathic and cell-adhesion assays were performed using GraphPad Prism version 7.0 (GraphPad Software Inc., San Diego, CA, USA). Statistical significance of differences between groups was determined using a one-way ANOVA with Tukey’s comparison of means as a *post-hoc* test. *P*-values < 0.05 were considered significant. Results presented in graphs are mean values ± 95% confidence intervals for cell death plus standard errors of the mean for cell adhesion of at least 1000 cells per group/experiment and from three independent experiments.

## Results

### Mouse immunization with mixed *Entamoeba* species induced higher and faster antibody response than single species

**Fig. 1:**
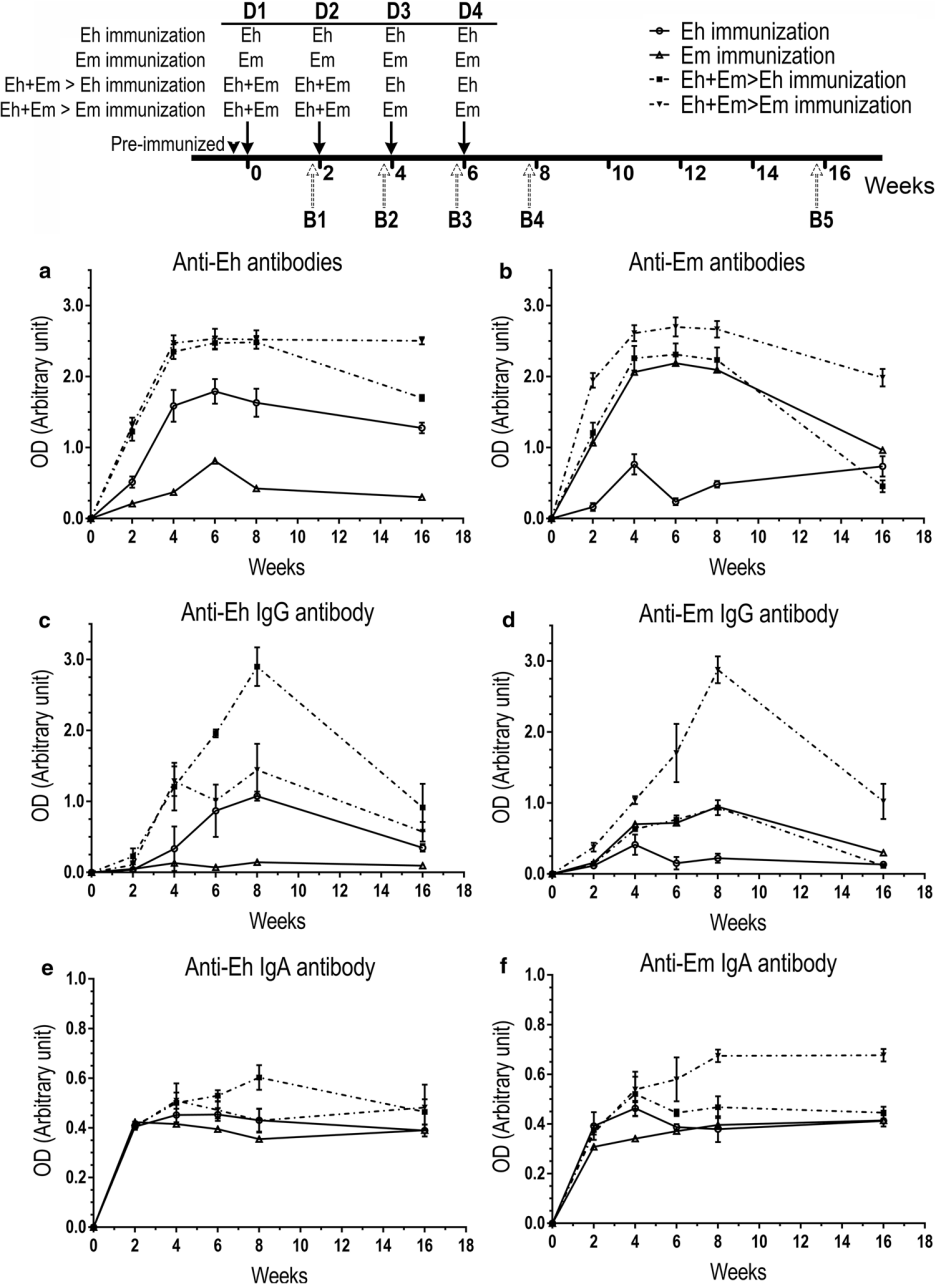
Mouse antibody response profiles to *E. histolytica* and/or *E. moshkovskii* trophozoites. Mice were immunized with trophozoite cells of *E. histolytica* and/or *E. moshkovskii*. Level of *E. histolytica* and *E. moshkovskii* total antibodies (**a**, **b**), species-specific IgG (**c**, **d**) and IgA (**e**, **f**) were measured by ELISA using goat anti-mouse IgG or IgA labeled with HRP as antibody tracker. B1–B4 represent numbers of serum collected 2 weeks after each 1st-4th immunization and B5 represents serum collected 8 weeks after the 4th boost. The results were presented as means of optical density (OD) ± standard error of the mean (SEM) from three independent experiments

Mouse immunization with *E. moshkovskii* trophozoite cells was shown to induce higher and faster total-antibody response than *E. histolytica*. Mice immunized with *E. histolytica* induced production of cross-*Entamoeba* species antibodies as *E. moshkovskii* (Fig. 1a, b). Compared to single species immunization, mice pre-challenged with mixed trophozoite cells of *E. histolytica* and *E. moshkovskii*, followed by either *E. histolytica* or *E. moshkovskii* cells, induced significantly higher levels of total antibodies and specific IgG to both species (Fig. 1a-d). Only the mice pre-immunized with mixed *E. moshkovskii* and *E. histolytica* cells were able to stimulate cross-species IgG (Fig. 1c, d). Species-specific IgG antibodies of mice immunized with the single *Entamoeba* species decreased sharply to the same level as the pre-immunized sera at 2 months after the last immunization. Levels of *E. histolytica-* and *E. moshkovskii*-specific IgG antibodies of mice immunized with mixed *Entamoeba* before individual species fell sharply at 2 months after the last boost, as seen in mice immunized with single *Entamoeba* species. However, these levels of *E. histolytica-* (Fig. 1c) and *E. moshkovskii-*specific IgG (Fig. 1d) remained about two-times higher than mice immunized with single *Entamoeba* species. Both *E. histolytica* and *E. moshkovskii* trophozoite cells induced a low level of cross-species IgA (Fig. 1e, f). Mice immunized with mixed *Entamoeba* species followed by *E. histolytica* stimulated the highest level of species-specific IgA; however, their levels decreased sharply after the last boost (Fig. 1e). Mice immunized with mixed *Entamoeba* species followed by *E. moshkovskii* stimulated a higher level of species-specific IgA than those immunized with *E. moshkovskii* cells alone (Fig. 1f). Furthermore, levels of *E. moshkovskii-*specific IgA antibodies remained high for at least 2 months after the last boost (Fig. 1f).

### Species-specific IgG antibodies promoted cytopathicity of *E. histolytica* toward CHO cells

**Fig. 2:**
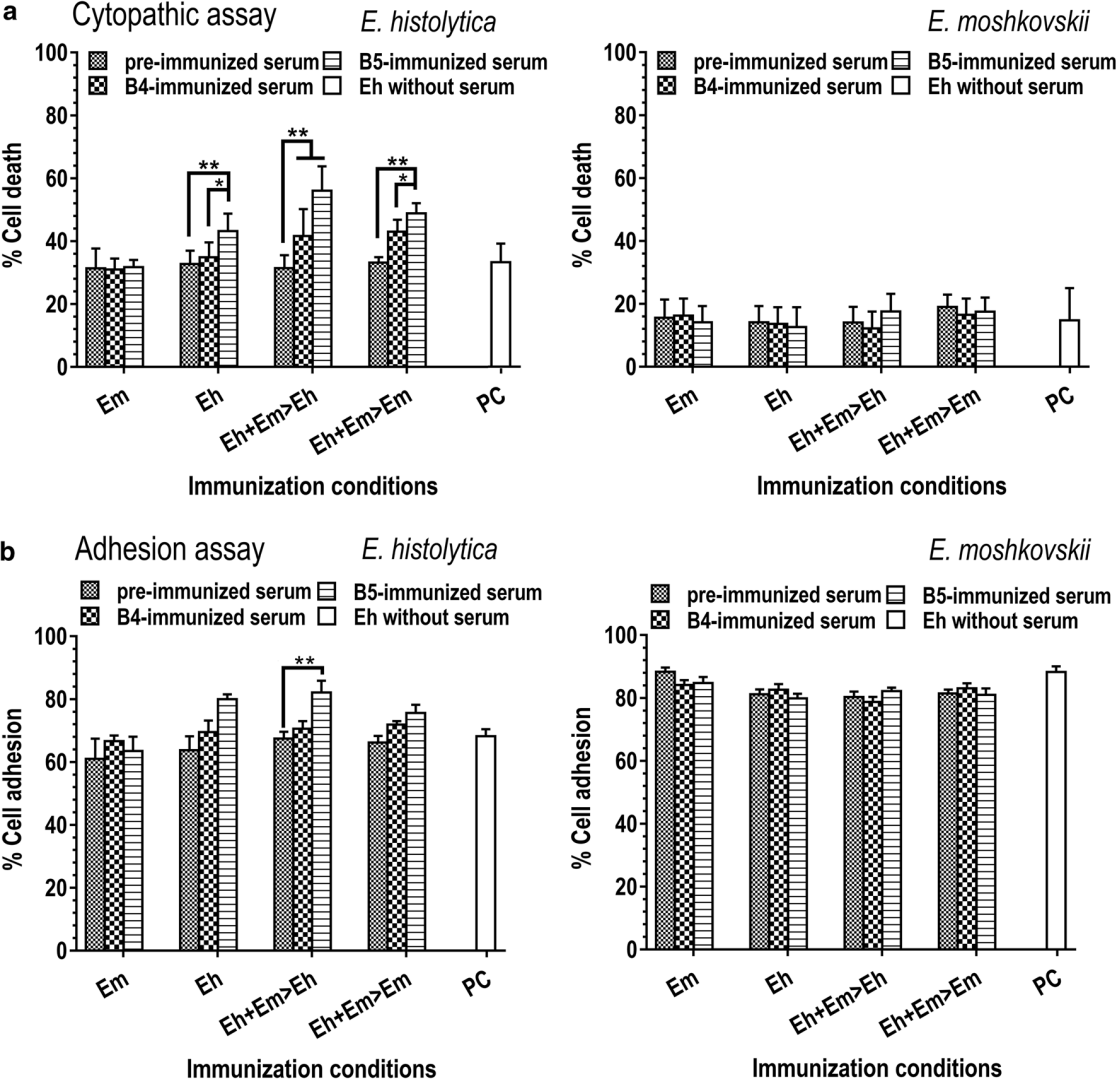
Cytopathic and adhesion assays of *E. histolytica* and *E. moshkovskii* with mouse sera. CHO cells were incubated with *E. histolytica* and *E. moshkovskii* trophozoite cells in the presence of mouse sera immunized with *E. histolytica* (Eh) or *E. moshkovskii* (Em) alone, mixed of *E. histolytica* and *E. moshkovskii* followed by *E. histolytica* (Eh + Em > Eh), and the mixed cells followed by *E. moshkovskii* (Eh + Em > Em). The effect of mouse sera on cytopathic activity and adhesion of *E. histolytica* and *E. moshkovskii* to CHO cells were determined by cell staining with propidium iodide (PI) and counting of PI positive and adhered CHO cells, respectively. PC is amoeba and CHO mixture without serum. B4 and B5 represent sera collected 2 weeks and after 4 times of trophozoite-cell immunization. Pre-immunized serum of each mouse was use as a negative control. The results are presented as percent cell death with 95% confidence interval from three independent experiments. *F*-values refer to ANOVA across all three challenges, asterisks indicate significant differences between challenges (Tukey’s *post-hoc* comparison), **P < 0.01 and *P < 0.05

**Fig. 3:**
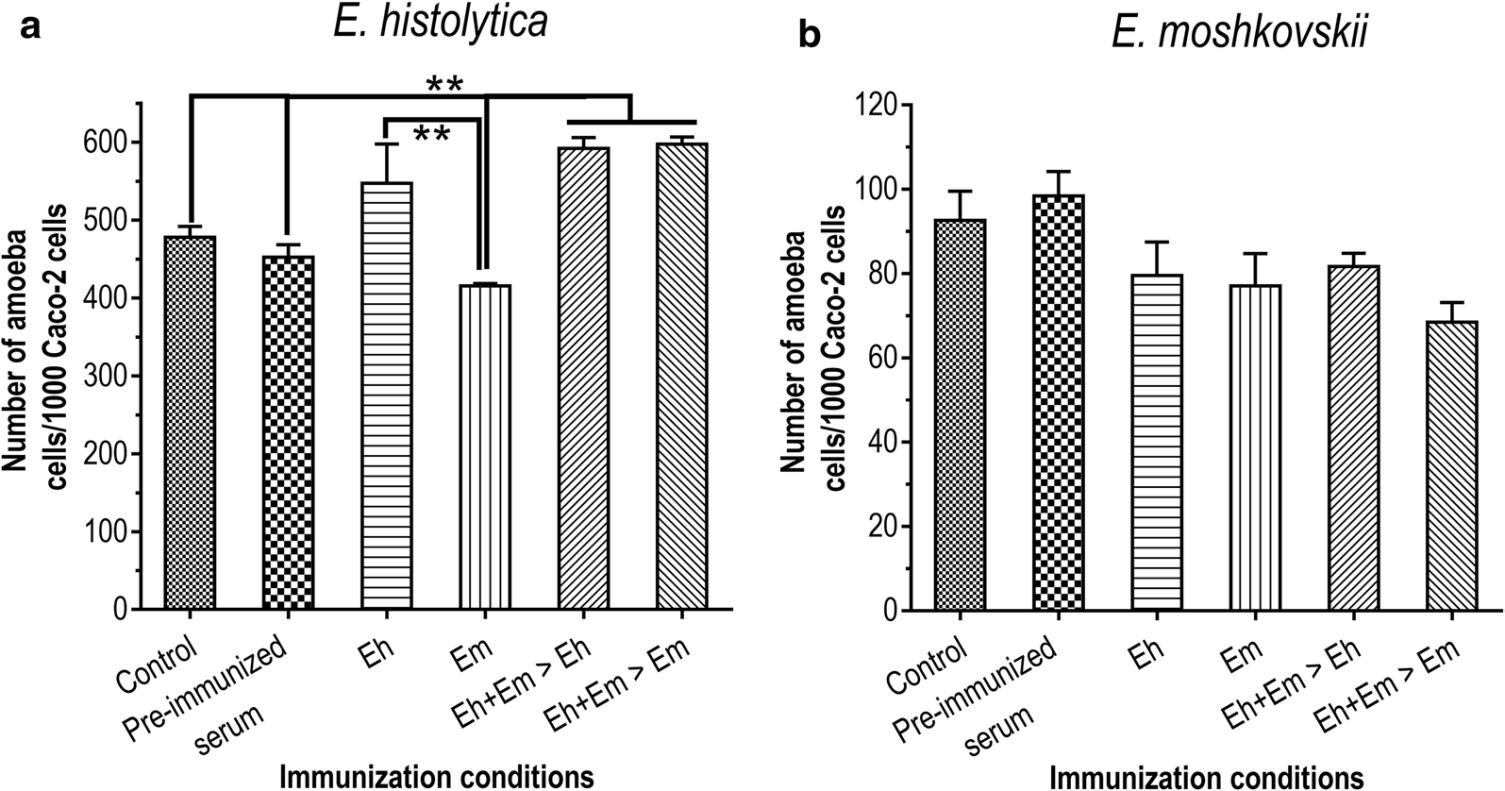
Adhesion of *E. histolytica* and *E. moshkovskii* to Caco-2 cells. Trophozoite cells of *E. histolytica* (**a**) and *E. moshkovskii* (**b**) were incubated with the Caco-2 human epithelial cell line in the presence of a serum of mice immunized with trophozoite cells, with immunization conditions as mentioned and pre-immunized serum as a control. After washing, trophozoite cells bound on Caco-2 cells were counted and represented as means of amoeba number per 1000 Caco-2 cells ± SEM from three independent experiments. Asterisks indicate significant differences between challenges, **P < 0.01. *Abbreviation*: SEM, standard error of the mean

CHO cells were challenged with *Entamoeba* cells pre-incubated with serum collected at 2 weeks (B4) and 2 months (B5) after dose-4 of *Entamoeba*-cell immunizations. The cytopathic ability of *Entamoeba* cells is represented as percent death with 95% CI of CHO cells. *Entamoeba histolytica* alone induced 33% (95% CI: 31.4–35.0%) of CHO-cell death (Fig. 2a, PC), which was close (ANOVA: *F*_(4,40)_ = 0.005, *P* = 0.99) to those of amoeba cells incubated with pre-immunized serum (33 ± 2.7%). *Entamoeba histolytica* cells incubated with the B4 and B5 sera of mice immunized with heterologous *Entamoeba* species followed *E. histolytica* (Eh + Em > Eh; ANOVA: *F*_(2,24)_ = 19.64, *P* = 0.01), *E. moshkovskii* (Eh + Em > Em; ANOVA: *F*_(2,24)_ = 35.68, *P* = 0.01) and the sera of *E. histolytica* (Eh; ANOVA: *F*_(2,20)_ = 7.53, *P* = 0.01) induced a significantly higher cytopathic ability of *E. histolytica* than cells pre-incubated with pre-immunized serum as well as the positive amoeba control without a serum (Fig 2a, cytopathic assay, *E. histolytica*). Numbers of *E histolytica* cells (68 ± 2.2%) were found to adhere to CHO cells. Most *E. histolytica* cells pre-incubated with immunized sera, especially B5 serum of the mice immunized with Eh + Em > Eh cells, were found to promote to the parasite adhesion to CHO cells (ANOVA: *F*_(2,11)_ = 6.8, *P* = 0.01) (Fig. 2, *E. histolytica*) and Caco-2 cells (ANOVA: *F*_(5,16)_ = 13.86, *P* = 0.01) (Fig. 3a).

Interestingly, *E. moshkovskii* showed cytopathic activity of 15 ± 3.4% (Fig. 2a, cytopathic assay, *E. moshkovskii*) and 88 ± 1.8% adhesion to CHO cells (Fig. 2b, adhesion assay, *E. moshkovskii*). However, there was no difference in cytopathic ability (ANOVA: *F*_(12,91)_ = 0.74, *P* = 0.70) and CHO cell adhesion (ANOVA: *F*_(12,99)_ = 1.61, *P* = 0.10) of *E. moshkovskii* in serum pre-treated and un-treated conditions. Immunized sera were tested further for their effect on amoeba adhesion to Caco-2 cells. Despite inhibition of *E. moshkovskii* adhesion to Caco-2 cells, there was no significant difference (ANOVA: *F*_(5,30)_ = 1.91, *P* = 0.12) in parasitic adhesion among the immunized, pre-immunized sera and serum un-treated amoeba (Fig. 3b).

### Cellular localization of mAb binding partners in *E. moshkovskii* and *E. histolytica* trophozoite cells

**Fig. 4:**
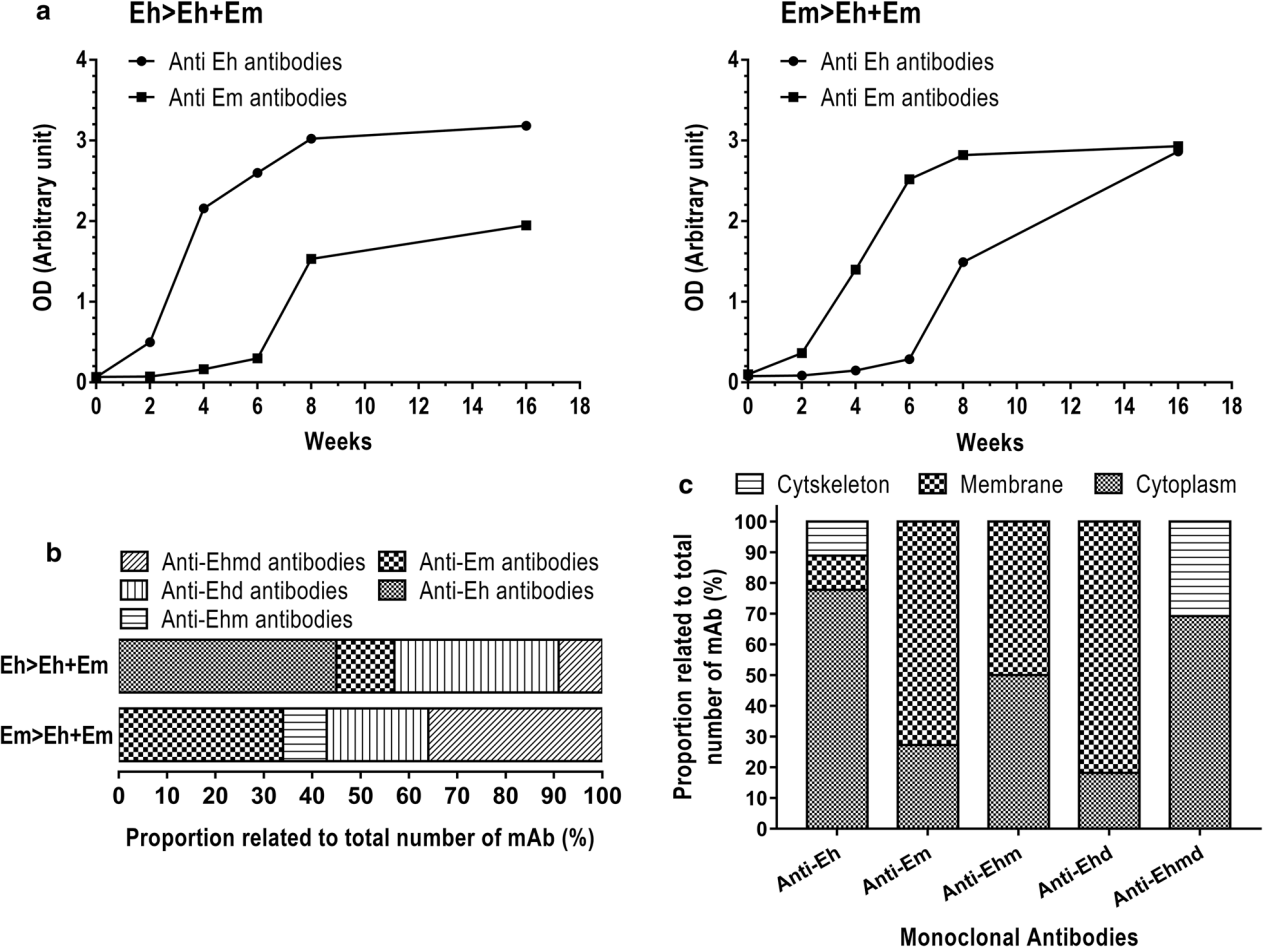
Proportion of *Entamoeba* species-specific mAbs and their specificities to cellular components. Mice were immunized with trophozoite cells of *E. histolytica* and *E. moshkovskii* followed by mixed cells of *E. histolytica* and *E. moshkovskii*, Eh > Eh + Em and Em > Eh + Em (**a**), respectively. The total antibody responses of the immunized mice were determined by ELISA using total lysate proteins of trophozoite cells of *E. histolytica* (solid circle) and *E. moshkovskii* (solid square) trophozoite cells. Proportions of mAbs specific to *E. histolytica* (Anti-Eh), *E. moshkovskii* (Anti-Em) and cross-species (Anti-Ehm, and Anti-Ehd, Anti-Ehmd) and their specificity toward cytoplasmic, membrane and cytoskeletal compartments of *E. histolytica* and *E. moshkovskii* are represented as % of mAbs (**b**, **c**)

**Fig. 5:**
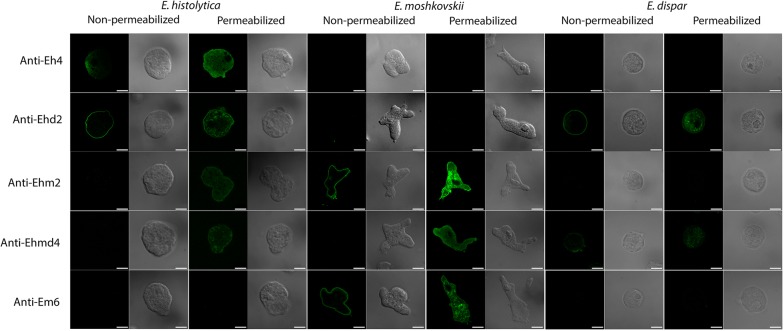
Cellular localization of mAb-recognition molecules. Non-permeabilized or Triton X-100 permeabilized (0.2%) trophozoite cells of *E. histolytica*, *E. moshkovskii* and *E. dispar* were stained with representative mAbs by immunofluorescent assay. The bound mAbs were tracked by fluorescein isothyocyanate (FITC)-conjugated goat anti-mouse IgG antibodies and observed under confocal microscopy (LSM 700; Carl Zeiss). *Scale-bars*: 10 µm

Mice were immunized with either *E. histolytica* or *E. moshkovskii* trophozoite cells and boosted with a cell mixture of *E. histolytica* and *E. moshkovskii* (Eh > Eh + Em, and Em > Eh + Em, respectively) to maximize the variety of *Entamoeba*-specific mAbs to study cellular localizations of the mAb-binding partners. Each set of immunized mice showed high antibody response to both *E. histolytica* and *E. moshkovskii*, respectively (Fig. 4a, b). In the immunization design with *E. histolytica* followed by mixed cells of *E. histolytica* and *E. moshkovskii*, 45% of mAbs were specific to *E. histolytica* (Eh), 34% to *E. histolytica* and *E. dispar* (Ehd), 9% to pan-human *Entamoeba*; *E. histolytica*, *E. moshkovskii* and *E. dispar* (Ehmd), and 12% to *E. moshkovskii* (Em) (Fig. 4a, Eh > Eh + Em). On the other hand, the immunization design with *E. moshkovskii* followed by mixed *Entamoeba* cells resulted in 34% of Em-specific mAb, 21% of Ehd mAbs, 36% of pan Ehmd mAbs, and 9% of Eh mAbs (Fig. 4b, Em > Eh + Em). These mAbs reacted against cytoplasmic, membranous and cytoskeletal fractions of *Entamoeba* cells. It was demonstrated that 78% of *E. histolytica*-specific mAbs targeted to cytoplasmic, 11% to cytoskeleton and 11% to membrane fractions (Fig. 4c). In contrast, 73% of *E. moshkovskii-*specific mAbs targeted membrane and only 27% toward cytoplasmic fractions. For cross-species mAbs, Ehm-specific mAbs equally targeted cytoplasm and membrane fractions of *E. histolytica* and *E. moshkovskii* trophozoite cells. Most of Ehd mAbs specifically directed to membrane (82%) and 18% to cytoplasmic fractions of *E. histolytica* and *E. dispar* cells. About 69% of pan-Ehmd mAbs were found to target the cytoplasm and 31% of them to cytoskeleton (Fig. 4c). An immunofluorescent assay was performed to confirm specificity and cellular localization of mAb-binding sites in *Entamoeba* trophozoite cells. Cells were directly reacted with a mAb to assess mAb binding at the outer membrane of the cells (non-permeabilized condition), while mAb access to plasma membrane and cytoplasmic components of the cells was performed through permeabilization using triton X-100 detergent (permeabilized condition). All species-specific mAbs bound specifically to the corresponding *Entamoeba* species (Fig. 5) as shown in the previous result (Fig. 4b). Abundant binding sites of Eh mAb (Fig. 5, Anti-Eh4) and pan-Ehmd mAb (Fig. 5, Anti-Ehmd4) were observed in the cytoplasm of *Entamoeba* trophozoite cells. In contrast, most binding sites of Ehd mAb (Fig. 5, Anti-Ehd2) and Em mAb (Fig. 5, Anti-Em6) were at the outer cell membrane and cytoplasm. Binding localization of Ehm- (Fig. 5, Anti-Ehm2) and Ehd-specific mAbs (Fig. 5, Anti-Ehd2) spanned from outer- and inner-plasma membrane and cytoplasm of the *Entamoeba* cells. In contrast, most binding partners of pan-human Ehmd mAb (Anti-Ehmd4) were associated with cytoplasmic components and were weakly detected at the outer membrane of the three *Entamoeba* species.

## Discussion

Both humoral and cell-mediated immune responses have been demonstrated to play roles in *E. histolytica* infection and disease manifestation [22]. However, few details exist on host immune response to *E. moshkovskii*. In the present study, we found that *E. moshkovskii* trophozoites induced species-specific antibodies in mice faster than *E. histolytica*, suggesting a difference in host immune response to these closely related species. Furthermore, mice pre-challenged with mixed trophozoite cells of *E. histolytica* and *E. moshkovskii* followed by single *Entamoeba* species produced species-specific IgG and IgA antibodies to both invasive *E. histolytica* and non-invasive *E. moshkovskii* faster and higher than the single species immunizations. These high responses among *Entamoeba*-specific antibodies may explain the ability of people in endemic areas to tolerate *Entamoeba* infection, where mixed infections of *Entamoeba* species are always common [23, 24]. On the other hand, a high level or titer of *E. histolytica*-specific IgG has been widely used as a diagnostic key for amoebic liver abscess (ALA), a severe infection outcome of *E. histolytica* [25]. Here, upon intra-peritoneal inoculation with *Entamoeba* trophozoite cells, we found that levels of *E. histolytica* and *E. moshkovskii*-specific IgG in mouse sera corresponded to doses/numbers of immunizations and dropped sharply after the last immunization. This result firstly reveals the association of a level of species-specific IgG of mice and the presence of these two *Entamoeba* species in the stomach. Measurement of serum IgG may be applied for monitoring intestinal infections by *E. histolytica* and *E. moshkovskii* trophozoites. Mice experienced with a *E. histolytica* and *E. moshkovskii* cell mixture prior to secondary immunization with either *E. histolytica* or *E. moshkovskii*, produced IgG antibodies to the latter species higher and faster than those mice immunized with single *Entamoeba* species. This may have resulted from the immunological memory of the mouse adaptive immune system [26]. IgA antibodies produced by plasma cells within the *lamina propria* serve as physical barrier to prevent intestinal mucus from adhesion and invasion of pathogens [27]. Antigens/proteins of *E. histolytica* were injected *via* the oral, intradermal, intramuscular and subcutaneous routes together with adjuvants to elicit strong IgA antibody-, cell-mediated responses and protection of the host [28]. In this study, without any adjuvant, mixed *E. histolytica* and *E. moshkovskii* trophozoite cells followed by *E. moshkovskii* were inoculated in the intraperitoneal route, and could elicit IgA antibody response of mice more than the mixed *E. histolytica* and *E. moshkovskii* cells followed by *E. histolytica* or with *E. moshkovskii* alone. Furthermore, the *E. moshkovskii*-specific IgA antibodies were produced faster and lasted longer than those with *E. histolytica*. This may be due to the strong immunogenicity of *E. moshkovskii* and corresponds to previous reports that *E. moshkovskii* was eliminated from the host faster than *E. histolytica* [17, 29].

Pathogenicity of *E. histolytica* trophozoites is mediated by parasite adhesion with gut mucin through parasitic Gal/GalNAc lectin [30] and actions of cysteine proteinases as mentioned previously. IgG antibody responses of patients have been shown to associate with infection susceptibility and pathogenicity and re-infection of *E. histolytica* [22]. In this study, sera of mice immunized with *E. histolytica* inversely promoted cytopathicity of *E. histolytica* towards CHO cells. Most sera of the *E. histolytica* immunized mice were found to significantly enhance parasitic adhesion to colonic Caco-2 cells. However, mice immunized with *E. histolytica* and *E. moshkovskii* cells followed by *E. histolytica* promoted parasite adhesion to CHO cells. It was likely that increased in adhesion and cytopathic activity of *E. histolytica* to these cells are associated with a level of specific IgG antibodies in the immunized sera with the exception of B5-immunized sera. It should be noted that B5 sera were collected two months after the last immunization, where a level of *E. histolytica* specific IgG antibodies have decreased. Therefore, there remains no explanation for the positive effect of these sera on adhesion and cytopathic activity of the parasite. However, it might be due to the sum of affinity/avidity of polyclonal antibodies found in the immunized sera after repeated immunizations. The present study also reflects a difference in susceptibility of tissue cells for *E. histolytica* infection. In parasitic *E. histolytica*, the role of specific IgG antibodies in pathogenicity is not clear; however, it has been suggested to depend on IgG subclasses. IgG2 and IgG4 have been reported as markers to follow-up ALA and recurrent infection of the parasite, respectively, whereas IgG1 and IgG3 were found to associate with parasite clearance by immune cells [25, 31]. Genetic background and family history have also been considered as factors contributing to difference in isotypes of *E. histolytica* specific IgG [25]. Antibody-mediated pathogenicity and disease severity known as antibody-dependent enhancement (ADE) has been exclusively studied in dengue virus and found to occur during the secondary infection with different virus serotypes [32, 33]. During ADE, virus recognition antibodies promote virus entry into dendritic cells and macrophages through their Fc receptor and suppress host immune response, resulting in survival and high viral load and severe disease manifestation of the host [33, 34]. It is possible that *E. histolytica*-specific IgG might promote pathogenicity of *E. histolytica* through ADE. For *E. moshkovskii*-specific IgG and IgA antibodies, despite having moderate to high antibody levels, there is no profound effect of the immunized sera on adhesion and pathogenicity of *E. moshkovskii* towards CHO cells as well as parasitic adhesion to human epithelial Caco-2 cells. It might imply an alternative host immune response and different pathogenicity of *E. moshkovskii* from its closely related species *E. histolytica.*

MAbs have been produced to *E. moshkovskii* and *E. histolytica* trophozoite cells and their characterizations revealed different localization of mAb binding targets in the virulent *E. histolytica*, non-virulent *E. moshkovskii* as well as non-pathogenic *E. dispar.* Most of the *E. histolytica* mAbs target to the cytoplasmic components, whereas most of the *E. moshkovskii* mAbs and cross-species (anti-Ehm and anti-Ehd2) and pan-human *Entamoeba* (anti-Ehmd) mAbs mostly target to the cell membrane. Although most *E. histolytica*-specific mAbs were shown to react to cytoplasm of the *E. histolytica* trophozoite, its membrane proteins, including Gal/GalNAc lectin, have been recognized by polyclonal antibodies in sera of immunized mice or humans. This would explain the enhancing effect of the immunized sera on host-cell adhesion and cytopathic abilities of the *E. histolytica* trophozoites. Compared to non-virulent *E. moshkovskii*, *E. histolytica* trophozoites might possess an evolutional design to conceal its surface molecules from the host immune system. It has been shown that *E. histolytica* possesses a thick and complicated cell surface containing lipopeptidophosphoglycan to protect the amoeba from the host complement system [35]. In addition, *E. histolytica* has been reported to modify its surface antigenic proteins in response to surrounding bacteria and growth conditions [36]. Furthermore, *E. histolytica* has been shown to cleave, translocate and release antibody-surface molecule complexes from the parasite cell surface by the parasite protease and actin rearrangement [37, 38]. These events were not observed in the non-pathogenic *E. dispar* [39]. Likewise, influenza virus causes reinfection by drifting its antigenic surface protein hemagglutinin, and further shifting of this protein also enables the virus to cause the cross-species infection [40, 41]. Modification of surface proteins in order to escape host immunity and prolong infection have also been reported in the sleeping sickness causative agent, *Trypanosoma brucei*, and the cattle protozoan parasite, *Babesia bovis* [42, 43].

Antigen variation is a crucial virulent feature that pathogens adopt to survive from the host immune system [44]. Here, we found that anti-Ehm2 mAb reacts to the inner membrane of *E. histolytica* trophozoite cells but is observed on the outer membrane of *E. moshkovskii* cells. Likewise, anti-Ehmd4 mAb binds to inner membrane and cytoplasmic components of *E. histolytica* and *E. moshkovskii*, trophozoites but is additional observed on the cell surface of non-pathogenic *E. dispar*. Therefore, it is likely that variation of mAb binding molecules might occur in these three closely related *Entamoeba* species. It has also been shown that *E. histolytica* has strain-specific antigens, which contribute to their virulence [45]. *Plasmodium falciparum* protozoans are the best model for altering its *var* gene family to escape from host immune recognition and prolong its infection [46].

## Conclusions

A mixed infection of *Entamoeba* stimulated a higher IgA and IgG antibody response of the host compared to multi-infection by a single species of human-infecting *Entamoeba*. *Entamoeba moshkovskii* induced the host antibody response faster and higher than *E. histolytica*. *Entamoeba-*specific anti-sera promoted host cell adhesion and cytopathicity of *E. histolytica*, but showed no effect to those activities of *E. moshkovskii*. However, functional roles of the induced antibodies as well as levels of mucosal IgA in the mouse models with amoebic dysentery, colitis and liver abscess need to be further elucidated. Characterization of *E. histolytica*, *E. moshkovskii*, cross-species and pan-*Entamoeba* mAbs revealed profound surface antigen manipulation and translocation to conceal or escape from host immune recognition by *E. histolytica* over non-virulent *E. moshkovskii* and non-pathogenic *E. dispar*. These findings may be useful for further vaccine development and better understanding and pathogenicity and host immune modulation by *E. histolytica* in the future.

## Abbreviations

MAb: monoclonal antibody
SDS-PAGE: sodium dodecyl sulfate-polyacrylamide gel electrophoresis
ELISA: enzyme-linked immunosorbent assay
HRP: horse radish peroxidase
HCl: hydrochloric acid
TMB: 3,3′,5,5′-tetramethylbenzidine
EDTA: ethylene diamine tetraacetic acid
RPMI: Roswell Park Memorial Institute
PBS: phosphate-buffered saline
BSA: bovine serum albumin
CHO: Chinese hamster ovary
PI: propidium iodide
ALA: amoebic liver abscess
FITC: fluorescein isothiocyanate
ADE: antibody dependent enhancement
IL: interleukin
IFN-γ: interferon gamma
TNF-α: tumor necrosis factor alpha
